# Association of Serum Pyridoxal-5′-Phosphate, Pyridoxal, and PAr with Colorectal Cancer Risk: A Large-Scale Case-Control Study

**DOI:** 10.3390/nu14122389

**Published:** 2022-06-09

**Authors:** Lei Xu, Yu-Jing Fang, Meng-Meng Che, Alinuer Abulimiti, Chu-Yi Huang, Cai-Xia Zhang

**Affiliations:** 1 Department of Epidemiology, School of Public Health, Sun Yat-sen University, Guangzhou 510080, China; xulei55@mail2.sysu.edu.cn (L.X.); chemm@mail2.sysu.edu.cn (M.-M.C.); abulimiti@mail2.sysu.edu.cn (A.A.); huangchy7@mail2.sysu.edu.cn (C.-Y.H.); 2 Department of Experimental Research, Sun Yat-sen University Cancer Center, State Key Laboratory of Oncology in South China, Collaborative Innovation Center for Cancer Medicine, 651 Dongfeng Road East, Guangzhou 510060, China; fangyj@sysucc.org.cn

**Keywords:** vitamin B_6_, pyridoxal-5′-phosphate, PAr, serum, colorectal cancer

## Abstract

Previous epidemiological studies have focused on the association of dietary vitamin B_6_ or circulating pyridoxal-5′-phosphate (PLP) with colorectal cancer risk. This study aimed to investigate the vitamin B_6_ in relation to colorectal cancer risk combining the biomarkers of PLP, pyridoxal (PL) plus PLP, and PAr (the ratio of 4-pyridoxic acid over the sum of PLP and PL). A large-scale hospital-based case-control study was conducted in Guangdong Province, China, which included 1233 colorectal cancer cases and 1245 sex and age frequency-matched controls. Serum PLP, PL, and 4-pyridoxic acid (PA) were detected with ultra-high-performance liquid chromatography–tandem mass spectrometry. Unconditional logistic regression models were used to assess the odds ratios (ORs) and 95% confidence intervals (95% CIs). Serum PLP and the sum of PLP and PL were inversely associated with colorectal cancer risk, while PAr was positively associated with colorectal cancer risk. Comparing the highest with the lowest quartile, the adjusted OR (95% CI) was 0.26 (0.20–0.33, *P*_trend_ < 0.001) for serum PLP, 0.51 (0.40–0.66, *P*_trend_ < 0.001) for serum PLP plus PL, and 2.90 (2.25–3.75, *P*_trend_ < 0.001) for PAr. Serum PLP and PAr had significantly stronger associations with colorectal cancer risk in the male group and smoking group. Our results supported the protective role of vitamin B_6_ in colorectal cancer risk among Chinese people. The positive association of PAr with colorectal cancer risk suggested the potential role of inflammation and oxidative stress in colorectal carcinogenesis.

## 1. Introduction

Colorectal cancer is the third most commonly diagnosed cancer and the second leading cause of cancer death in the world, with more than 1.9 million cases and 935,000 deaths estimated in 2020 [1]. Reducing the burden of colorectal cancer could be achieved through nurturing healthy lifestyles and proper dietary habits [2]. However, dietary guidelines for the prevention of colorectal cancer are insufficient due to the complicated effects of some nutrients.

Vitamin B_6_ involves over 100 enzymatic responses including carbohydrate metabolism, glycogenolysis, and lipid/homocysteine metabolism [3]. In vivo and in vitro experiments have demonstrated the antitumor properties including suppressing tumorigenesis, cell proliferation, and angiogenesis [4,5]. However, epidemiological studies investigating the relationship between dietary vitamin B_6_ intake and colorectal cancer have drawn inconsistent conclusions [6,7,8]. Measurement errors might explain, at least partially, the inconsistent results for dietary assessment [9]. Several combinations of circulating biomarkers can provide information on the nutritional status or function [10]. Thus, direct measurement of vitamin B_6_ biomarkers could further provide complementary information on exposure.

Pyridoxal-5′-phosphate (PLP), a primary active form of vitamin B_6_, is commonly used as a biomarker of vitamin B_6_ status [11,12]. The results from prior epidemiological studies investigating the relationship between circulating vitamin B_6_ and colorectal cancer have been slightly inconsistent [13,14,15,16,17,18,19]. Most, but not all, studies have reported an inverse association of plasma PLP with colorectal cancer risk [14,15,16,18]. Pyridoxal (PL), the dephosphorylated form of PLP, has been regarded to be the ultimate transport form of vitamin B_6_ [20]. Degraded PLP in serum has been recovered as PL in room temperature conditions [21].The sum of the PLP and PL concentrations was stable, and therefore, it could correct for partial loss of PLP during non-optimal sample handling [11]. To some extent, the sum of PL and PLP could represent the level of vitamin B_6_ in blood samples as the supplementary index of PLP. So far, no studies have investigated the relationship between vitamin B_6_ status and colorectal cancer risk with this index.

Dephosphorylation of PLP has been converted to PL, and finally metabolized to the metabolite 4-pyridoxic acid (PA), which is the major channel of vitamin B_6_ metabolism in humans [22]. PAr, calculated by the ratio of PA versus the sum of PLP and PL, has been proposed to be a novel biomarker. PAr has been shown to increase when the key PLP-catabolizing enzymes were upregulated in aldehyde and oxidative stress [23]. In addition, some inflammatory markers could account for approximately 90% of the variance of PAr, which suggested that PAr could indicate the catabolism of vitamin B_6_ during inflammation [24]. The novel index has been used to estimate the association of vitamin B_6_ catabolism with cancers, such as lung cancer [24,25] and pancreatic cancer [26]. To date, only two studies have reported the association of PAr with colorectal cancer risk, but the results were controversial [17,27]. Therefore, more studies are needed to address the question.

In this context, this study aimed to evaluate the association of vitamin B_6_ with colorectal cancer risk among Chinese people, using diverse biomarkers to reflect vitamin B_6_ status and catabolism level. We hypothesize that vitamin B_6_ status is inversely associated with colorectal cancer risk, while vitamin B_6_ catabolism is positively associated with colorectal cancer risk.

## 2. Materials and Methods

### 2.1. Study Subjects

This ongoing study began in July 2010 for the purpose of examining the relationship between lifestyle factors and colorectal cancer risk in Guangdong, China and has been previously described in detail [28]. Briefly, potential cases aged 30–75 years were consecutively recruited from the surgical units of the Sun Yat-sen University Cancer Center in Guangzhou, China. Cases were histologically diagnosed with primary colorectal cancer within 3 months, who were Guangdong natives or residents having lived in Guangdong for at least 5 years. The patients were excluded if they were diagnosed with other cancers or they could not understand or speak Mandarin/Cantonese. From July 2010 to May 2021, a total of 3174 cases were identified and 2833 eligible cases completed the interview with a response rate of 89.26%. Among them, 1303 blood samples were available. We excluded those with abnormal measurements before matching with controls, resulting in 1233 cases included in the final analysis.

The control subjects, free of any cancers ever, were frequency matched to cases by age (with 5-year intervals) and sex. One of the control groups was selected from the Departments of Ophthalmology, Plastic and Reconstructive Surgery, Vascular Surgery, Otolaryngology, Orthopedics, and Microsurgery in the First Affiliated Hospital of Sun Yat-sen University during the same period as the cases. In total, 2283 hospital-derived controls were identified and 2044 controls were interviewed with a response rate of 89.53%. Among them, 1841 blood samples were available. The other control group was recruited from the same community as the cases by the various channels. A total of 934 community-derived controls with available blood samples completed the interview. After excluding abnormal measurement data and matching with cases, a total of 1245 controls were included in the final analysis.

The study was conducted in accordance with the ethical standards formulated in the 1964 Declaration of Helsinki and authorized by the Ethical Committee of Public Health, Sun Yat-sen University (approval number 2020-116). All participants signed an informed consent form before the interview.

### 2.2. Data Collection

Trained interviewers conducted a face-to-face interview using a well-structured questionnaire. The following information was collected from study subjects: socio-demographic characteristics, lifestyle habits (active and passive smoking, alcohol drinking, and physical activity), body height and weight, and history of cancer in first-degree relatives. Detailed information regarding use of nutritional supplements was also obtained. For the female subjects, information on age at menarche and menopausal status were collected additionally. All participants were asked to give full detailed information on their habitual dietary intake during the past year before the diagnosis using the validated food frequency questionnaire [29]. The average daily nutrient intake was calculated by multiplying the frequency of each food consumption, their corresponding portion size and nutrient content for each food item, based on the Chinese Food Composition Table [30]. Body mass index (BMI) was calculated by the ratio of weight (kg) dividing the square of height (m^2^). In this study, a regular smoker was defined as someone who had smoked at least one cigarette for more than 6 months continuously in their daily life. A regular drinkers was defined as someone who consumed alcohol at least once a week over the past year. Occupational activity was categorized into non-working, sedentary, light occupation, moderate occupation, and heavy occupation activities. Metabolic equivalent (MET) value of housework and recreational activities was estimated according to the Compendium of Physical Activities [31,32]. The MET-hours/week during the previous 12 months was calculated as follows: number of days per week × number of hours per day × MET for a specific type of activity = MET-hours/week.

### 2.3. Biochemical Measurement

Approximately 5 mL fasting venous blood was drawn from each participant on the second day after admission to the hospital. Participants had not received any treatment before blood collection. The blood samples were transported in a sealed box filled with dry ice to the laboratory within 1 h. As soon as arrival at the laboratory, each blood sample was centrifuged at 3000 rpm for 10 min at 4 °C. Each serum sample was isolated and aliquoted to eight tubes, and then stored at −80 °C in a freezer until analysis.

Serum vitamin B_6_ species, including PLP, PL, and PA, were detected by high-performance liquid chromatography–tandem mass spectrometry (HPLC–MS/MS). The detection method was modified according to the previously published methods [33,34,35]. The detailed process of measurement was as follows. An aliquot of 100 μL serum sample was taken into a 2 mL centrifugal tube and spiked with 10 μL deuterated internal standards containing 50 nmol/L each of d_3_-PLP and d_3_-PL. For protein precipitation, 400 μL methanol (HPLC grade) was added to the serum sample. The mixture was vortexed for 2 min, left for 20 min at room temperature, and finally centrifuged at 15,000 rpm for 15 min. Approximately 400 μL of supernatant was pipetted to the new centrifugal tube and evaporated under nitrogen flow at room temperature. The residue was resolved with 100 μL of mobile phase A (water containing 0.2% acetic acid) and completed with 1% ascorbic acid. The analytical sample was injected into an Agilent 1290 UHPLC system (Agilent, Santa Clara, CA, USA) equipped with an Agilent poroshell 120 CE C18 column (3 mm × 100 mm, 2.7 μm) and an Agilent integrated precolumn (3 mm × 5 mm, 2.7 µm). Mobile phase A was water containing 0.2% (*v*/*v*) acetic acid and mobile phase B was acetonitrile. The final mobile phase gradient started with 98% phase A, and then was followed according to the timetable: 0.5–5 min (95% A and 5% B), 6–9 min (2% A and 98% B), and 10–13 min (98% A and 2% B). All gradient steps were linear with a constant flow rate of 0.3 mL/min. The samples were analyzed using an Agilent 6495 triple quadrupole mass spectrometer with ESI source (Agilent, USA). Quantitative analysis was performed in positive mode (ESI+) using multiple reaction monitoring (MRM). The mass transitions (*m*/*z*) were 248.1–150 for PLP, 168.1–150 for PL, 184.1–148 for PA, 251.1–153 for d_3_-PLP, and 171.1–153 for d_3_-PL.

A similar number of cases and controls were analyzed in the same detected batch. The average recoveries of all serum B_6_ vitamins were in the range of 85–118%. Mean inter-batch coefficients of variation were 8.1% for PL, 11.4% for PLP, and 10.1% for PA. The limits of detection (LOD) values for this method were 1.0 nmol/L for PL, 0.5 nmol/L for PLP, and 0.2 nmol/L for PA.

### 2.4. Statistical Analysis

The differences in demographic characteristics between cases and control subjects were examined utilizing the Student’s *t*-test or Wilcoxon signed-rank test for the continuous variables, and the χ2 test for the categorical variables. The measured serum levels of each vitamin B_6_ biomarker below the LOD were determined as LOD/2. Selected nutrient intake was adjusted by the energy residual method and the concentrations of serum PLP and the sum of PL plus PLP were categorized into quartiles (Q1–Q4) based upon the distribution among the controls for males and females, separately. The value of PAr was calculated as the ratio of PA to PL plus PLP.

An unconditional logistic regression was used to estimate odds ratio (OR) and 95% confidence intervals (CI) for the analysis of the relationship between serum concentrations of each vitamin B_6_ biomarker and the colorectal cancer risk after adjusting for potential confounders. Potential confounders were selected based on the discrepancies by comparing the baseline characteristics of the cases with the controls. In the multivariable analysis, we controlled for age, sex, BMI, education level, income level, marital status, smoking status, occupational activity, and MET-h/week. Given some nutrients and one-carbon metabolism-related nutrients with possible links to both exposure and outcome, we further adjusted for multivitamin supplement use, total energy, protein, vitamin B_2_, folate, vitamin B_12_ and methionine intake. For female participants, age at menarche was additionally adjusted. Liner trends were evaluated by entering categorical variables as continuous variables in the logistical regression model.

Restricted cubic spline (RCS) was used to examine the dose–response relationship between serum vitamin B_6_ and colorectal cancer. We fitted the model with 4 knots (located at 5th, 35th, 65th, and 95th) of serum PLP, 3 knots (located at 10th, 50th, and 90th) of serum PL plus PLP and PAr according to Akaike information criteria [36]. P for nonlinearity was calculated by Wald test.

A stratified analysis by sex was conducted. Moreover, smoking could influence the level of circulating PLP [37], thus, a stratified analysis by smoking status was also conducted. The multiplicative term of each vitamin B_6_ biomarker and sex or smoking status were fitted in unconditional logistic regression, respectively, to examine their interactions. Subgroup analysis by cancer site was also conducted. The case only analysis was used to examine the heterogeneity between colon and rectal cancer. The different subtypes were used as the dependent variable and each serum vitamin B_6_ biomarker was entered as an independent variable in logistical regression model.

Data analysis was performed with SPSS 21.0. (IBM Corporation, Armonk, NY, USA) and R version 4.0.5. *p* values < 0.05 were considered statistically significant.

## 3. Results

Among the 1233 colorectal cancer cases, 762 were diagnosed with colon cancer and 471 were diagnosed with rectal cancer. The demographic characteristics of the cases and controls are summarized in Table 1 As compared with the control subjects, a greater proportion of the cases were married and regular smokers. The cases had lower BMI, less education, higher income, fewer household and leisure activities, and fewer heavy occupation activities than the controls. In the female subgroup, cases tended to have an earlier age at menarche.

As presented in Table 2, dietary intake of total energy and energy-adjusted vitamin B_2_, folate, vitamin B_12_, and methionine levels were significantly lower in the cases than in control subjects. Serum levels of PLP, and PL plus PLP were significantly lower in the cases as compared with the controls.

Serum levels of PLP and the sum of PL and PLP were significantly inversely associated with the risk of colorectal cancer. Comparing the highest with the lowest quartile, the adjusted ORs (95% CI) were 0.26 (0.20–0.33, *P*_trend_ < 0.001) for serum PLP and 0.51 (0.40–0.66, *P*_trend_ < 0.001) for PL plus PLP. Serum PAr values were significantly associated with an increased risk of colorectal cancer, with an adjusted ORs (95% CIs) of 2.90 (2.25–3.75, *P*_trend_ < 0.001) for the highest versus the lowest quartile (Table 3).

**Figure 1:**
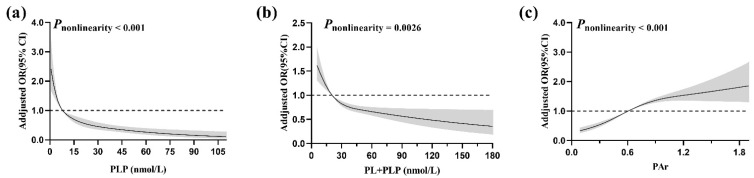
Relationships between serum PLP (**a**), PL plus PLP (**b**), PAr (**c**) levels and colorectal cancer risk by the restricted cubic spline model. The models were adjusted for age, sex, BMI, education level, income level, marital status, smoking status, occupational activity, MET-h/week, multivitamin supplement use, protein intake, total energy intake, vitamin B_2_ intake, folate intake, vitamin B_12_ intake, and methionine intake. The median of individual biomarker is the reference. The shaded areas represent 95% CI. PL, pyridoxal; PLP, pyridoxal 5′-phosphate; PA, 4-pyridoxic acid; PAr, PA/(PL + PLP) ratio.

The RCS analysis showed that there were significant nonlinear associations of serum PLP, the sum of PL and PLP, and PAr with colorectal cancer risk. Colorectal cancer risk decreased rapidly with increased levels of serum PLP until around 15 nmol/L, and then flattened out. The sum of PL and PLP had a similar L-shaped association with colorectal cancer risk. The risk of colorectal cancer increased rapidly initially, then, varied with a relatively slow increment beyond a PAr value of 1.0 (see Figure 1).

A sex-stratified analysis showed similar inverse associations between serum PL plus PLP and colorectal cancer risk in both sexes, but the inverse association between serum PLP and colorectal cancer risk was stronger in males than females (*P*_interaction_ = 0.001). Similarly, the positive association of PAr with colorectal cancer risk was stronger in males than in females (*P*_interaction_ = 0.001) (Table 4).

In the stratified analysis by smoking status, no significant interaction was found between serum PL plus PLP and colorectal cancer risk (*P*_interaction_ = 0.947). However, the inverse association between serum PLP and colorectal cancer risk was more evident in regular smokers than in never smokers (*P*_interaction_ = 0.008). The positive association between PAr and colorectal cancer risk was more apparent among regular smokers (*P*_interaction_ = 0.018) (Table 4).

A subgroup analysis by cancer site showed that serum PLP, and PL plus PLP levels were inversely associated with the risk of both colon cancer and rectal cancer (*P* for heterogeneity > 0.05). Similar strength positive associations of PAr values with colon and rectal cancer were observed (*P* for heterogeneity = 0.180) (Table 5).

## 4. Discussion

This study aimed to investigate the association of serum vitamin B_6_ status and vitamin B_6_ catabolism with colorectal cancer risk. The results showed that serum PLP and the sum of PL plus PLP level were inversely associated with colorectal cancer risk, whereas a higher PAr value was associated with an increased risk of colorectal cancer. The observed associations were more pronounced in males and regular smokers.

Our observation of an inverse association between serum PLP and colorectal cancer risk is consistent with some previous studies. A meta-analysis published in 2010 containing four nested case-control studies found that higher plasma or serum PLP was related to lower colorectal cancer risk [38]. The nested case-control study from the European Prospective Investigation into Cancer and Nutrition (EPIC) cohort reported that plasma PLP was inversely associated with colorectal cancer risk [18]. By contrast, one nested case-control study within the Northern Sweden Health and Disease Study observed no significant association between plasma PLP and colorectal cancer risk [17]. One case-control study from Taiwan, China, including 168 cases and 188 controls, also observed null association [19]. The non-significant associations might be due to relatively small sample sizes, which are more prone to obtaining a null association.

To date, no study has reported on the association between the sum of serum PL plus PLP concentration and colorectal cancer risk. All the vitamin B_6_ species, including PL, pyridoxamine, pyridoxine, and their respective phosphorylated forms, can convert into PLP to play a primary physiological role [22]. Moreover, PL also serves as an available source of vitamin B_6_ to meet possible increased metabolic demands [20]. Thus, the combination of PL and PLP levels could account for the availability of vitamin B_6_. The results from serum PL plus PLP could further confirm the relationship between available vitamin B_6_ status and colorectal cancer risk. Of course, our observation of the inverse association of serum PL plus PLP concentration with colorectal cancer risk needs to be confirmed in further studies.

Our study showed a significant positive association between serum PAr and colorectal cancer risk. Consistent with our results, one prospective study in Norway found that a higher plasma PAr value at baseline was positively associated with colorectal cancer risk [27]. However, a nested case-control study from Sweden including 613 cases and 1190 matched controls reported that the value of PAr tended to be positively associated with tumor progression rather than initiation [17]. These studies were both conducted in Nordic countries where dietary patterns, climate environment, and races differed greatly from the Asian countries.

The RCS analysis suggested a nonlinear relationship between serum PAr and colorectal cancer risk. A nested case-control study in Sweden found a linear positive association of plasma PAr with colorectal cancer risk [17]. Additionally, our study observed an approximate L-shaped relationship between serum PLP and colorectal cancer risk, whereas the abovementioned study in Sweden showed a significant U-shaped association [17]. Overall, the results of dose-response analyses from different studies have concluded that lower serum PLP, at least lower than median, were correlated with an increased risk of colorectal cancer. However, whether PLP could additionally reduce colorectal cancer risk after above sufficiency must be further verified.

One of the antitumor mechanisms of vitamin B_6_ might involve its anti-inflammation effect [39]. Chronic inflammation has generally been associated with the development of colorectal cancer [40]. The inflammatory process is closely related to oxidative and aldehyde stress [41]. In response, some enzymes involved in irreversible conversion of PL to PA are upregulated, which produces an increased PAr value [23]. It has been reported that circulating PLP was inversely associated with inflammation, which reflected the potential protective role of vitamin B_6_ in inflammation [42,43]. Decreased circulating PLP, as the denominator of the PAr, might also contribute to the increment of PAr. In addition, PLP also plays a critical role in one-carbon metabolism ensuring the balanced process of DNA synthesis, repair, and methylation, which may contribute to the initiation of colorectal cancer [44,45].

Our study revealed that serum PLP and PAr were differentially associated with colorectal cancer risk according to sex. However, a nested case-control study from Sweden found no interaction between sex and plasma PLP or PAr [17]. The exact mechanism of the sex difference is uncertain. The more significant associations of PLP and PAr with colorectal cancer risk observed in males may be related to sex hormones. Higher testosterone level was associated with increased colorectal cancer risk in men [46]. Additionally, a higher intracellular PLP level has the potential to decrease the transcriptional activation of the androgen and progesterone receptors [47], which could relate to the depletion of PLP and increasing the catabolism of vitamin B_6_.

Stratification by smoking status suggested that the significant association between serum PLP level or PAr and colorectal cancer risk was more apparent among regular smokers. However, one previous study did not find a significant modifying effect of smoking on the association between serum PLP and colorectal cancer risk [13]. A large number of reactive oxygen species contained in tobacco smoke could have induced the oxidative stress which contributed to the tumor formation and development [48,49]. The antioxidant defense of PLP [50,51] in regular smokers could result in the more significant association with colorectal cancer. Additionally, the inflammatory response induced by smoking could increase the PAr value [49], which produced a more evident association of PAr with colorectal cancer risk in regular smokers.

No significant heterogeneity was found in the association of serum vitamin B_6_ biomarkers with colon and rectal cancer risk. Our previous study on the association between dietary vitamin B_6_ intake and colorectal cancer risk observed similar results [7]. Two nested case-control studies from Sweden [17] and EPIC [18] also observed null differences between plasma PLP level or PArvalue with colon or rectal cancer risk. However, a nested case-control study from Finland reported a significant association of serum PLP with colon cancer only [13]. The null associations could be ascribed to small sample sizes after the split to subgroups of colon and rectal cancer cases, which included 151 and 126 matched case-control pairs each.

To the best of our knowledge, this is the first study to examine the association between serum PAr and colorectal cancer risk in Chinese people. Other strengths of this study include the relatively larger sample sizes and simultaneous detection of serum PLP, PL, and PA using HPLC–MS/MS. Our study has some limitations. First, selection bias is inevitable due to hospital-based case-control study design. The patients were selected from the Sun Yat-sen University Cancer Center, the biggest cancer center in Southern China. However, the clinic characteristics of the recruited patients from the hospital resemble other patients in mainland China [52]. Second, a single measurement may not represent the long-term circulating levels of biomarkers. However, a prior prospective study estimated within-person variability among 367 participants who donated two blood samples approximately 10 years apart and observed no considerable change of PLP level [53]. It suggested that blood PLP could reflect the long-term and stable exposure of vitamin B_6_. Finally, the serum PLP concentrations of most participants in our study were lower than those reported in other previous studies [13,14,15,16,17,18,27]. But a nested case-control study within four large cohorts including 208 pancreatic cancer cases and 623 controls reported a geometric mean plasma PLP concentration of 10.6 nmol/L for controls [54], of which result was close to ours (geometric mean is equal to 10.08 nmol/L). The different detection ranges in different studies could be ascribed to different levels of vitamin B_6_ intake, the decomposition of PLP in serum samples [21], and methodological variances. It was noted that our detection coefficients of variations were relatively low (below 15%), which indicated that the serum level of PLP detected in cases and controls simultaneously using the same method had little impact on the estimation of OR value.

## 5. Conclusions

In conclusion, our study found that serum PLP and the sum of PL plus PLP were inversely associated with colorectal cancer risk in a unique Chinese population, which expanded the scope of the applicable population that supported the protective role of vitamin B_6_ in colorectal cancer risk. We also added to the limited evidence that PAr was associated with an elevated colorectal cancer risk, suggesting the potential role of inflammation and oxidative stress captured by elevated PAr in colorectal carcinogenesis. Further prospective studies are warranted to confirm our findings.

## Figures and Tables

**Table 1:** The demographic factors of colorectal cancer cases and controls.

Characteristics	Cases (*n* = 1233)	Controls (*n* = 1245)	*P*
Age (years) (mean ± SD)	56.71 ± 10.23	56.50 ± 11.00	0.624
Sex (*n*, %)			0.904
Male	675 (54.74)	678 (54.46)	
Female	558 (45.26)	567 (45.54)	
BMI (kg/m^2^) (mean ± SD)	22.34 ± 3.05	23.66 ± 3.24	<0.001
Marital Status (*n*, %)			0.001
Married	1166 (94.57)	1136 (91.24)	
Unmarried/divorces/widowed	67 (5.43)	109 (8.76)	
Residence (*n*, %)			0.107
Urban	795 (64.48)	841 (67.55)	
Rural	438 (35.52)	404 (32.45)	
Education level			0.003
Primary school or below	398 (32.28)	362 (29.08)	
Secondary school	347 (28.14)	335 (26.91)	
High School	303 (24.57)	291 (23.37)	
College or above	185 (15.01)	257 (20.64)	
Occupation			0.765
Administrator/other white-collar worker	174 (14.11)	188 (15.10)	
Blue-collar worker	270 (21.90)	274 (22.01)	
Farmer/others	789 (64.99)	783 (62.89)	
Income (Yuan/month) (*n*, %)			<0.001
<2000	180 (14.60)	378 (30.36)	
2001–5000	389 (31.55)	299 (24.02)	
5001–8000	368 (29.85)	286 (22.97)	
>8001	296 (24.00)	282 (22.65)	
Regular smokers (*n*, %)	325 (26.36)	270 (21.69)	0.010
Regular drinker (*n*, %)	209 (16.95)	228 (18.31)	0.399
First-degree relative with cancer (*n*, %)	162 (13.14)	152 (12.21)	0.487
Multivitamin supplement user (*n*, %)	38 (3.08)	57 (4.58)	0.059
Occupational activity (*n*, %)			<0.001
Non-working	153 (12.41)	162 (13.01)	
Sedentary	328 (26.60)	359 (28.84)	
Light occupation	327 (26.52)	338 (27.15)	
Moderate occupation	233 (18.90)	141 (11.32)	
Heavy occupation	192 (15.57)	245 (19.68)	
Household and leisure activity (MET-h/week) (mean ± SD)	33.47 ± 27.90	38.69 ± 30.75	
			<0.001
Age at menarche (years) (mean ± SD)	14.70 ± 3.00	15.29 ± 2.13	<0.001
Menopausal status *			0.322
Premenopausal	162 (29.03)	180 (31.75)	
Postmenopausal	396 (70.97)	387 (68.25)	
Cancer site			
Colon	762 (61.80)	-	
Rectal	471 (38.20)	-	

MET, metabolic equivalent task. Continuous variables were evaluated using *t* tests or Wilcoxon rank-sum tests. Categorical variables were evaluated using χ2 tests. * Among female subgroup.

**Table 2:** Selected dietary factors and serum concentrations of vitamin B_6_ biomarkers between colorectal cancer cases and controls.

	Cases (*n* = 1233)	Controls (*n* = 1245)	*P*
	Median (25th, 75th)	Median (25th, 75th)	
Dietary intake ^a^			
Protein (g/day)	62.38 (55.14–71.55)	63.87 (55.61–71.74)	0.145
Vitamin B_2_ (mg/day)	0.83 (0.68–0.95)	0.87 (0.72–1.01)	<0.001
Vitamin B_6_ (mg/day)	0.82 (0.70–0.95)	0.85 (0.73–0.98)	<0.001
Folate (μg/day)	206.58 (178.36–240.93)	218.75 (185.61–256.50)	<0.001
Vitamin B_12_ (μg/day)	1.64 (1.15–2.32)	1.87 (1.34–2.49)	<0.001
Methionine (mg/day)	1163.17 (1007.82–1364.59)	1206.14 (1031.08–1374.22)	0.028
Total energy (kcal/day)	1501.42 (1232.13–1832.94)	1425.65 (1189.90–1724.46)	<0.001
Serum vitamin B_6_ biomarkers			
PLP (nmol/L)	6.20 (3.33–11.16)	10.06 (5.51–18.67)	<0.001
PL (nmol/L)	11.76 (8.00–11.76)	10.60 (7.27–16.48)	0.001
PL plus PLP (nmol/L)	19.11 (12.84–27.33)	22.37 (15.44–35.35)	<0.001
PA (nmol/L)	12.86 (9.90–18.87)	12.20 (8.58–17.24)	0.005
PAr	0.67 (0.48–0.96)	0.53 (0.36–0.79)	<0.001

^a^ Consumption was adjusted for total energy intake using the regression residual method. PLP, pyridoxal 5′-phosphate; PL, pyridoxal; PA, 4-pyridoxic acid; PAr: PA/(PL + PLP) ratio.

**Table 3:** Odds ratios (ORs) and 95% confidence intervals (95% CIs) of colorectal cancer according to quartiles of serum PLP, PL plus PLP, and PAr values.

	Q1	Q2	Q3	Q4	*P*_trend_
PLP					
Median (nmol/L)	3.26	7.45	13.05	28.68	-
Cases/controls	562/311	293/312	253/312	125/310	-
Crude OR (95% CI)	1.00	0.52 (0.42–0.64)	0.45 (0.36–0.56)	0.22 (0.17–0.29)	<0.001
Adjusted OR_1_ (95% CI) ^a^	1.00	0.55 (0.45–0.69)	0.49 (0.39–0.61)	0.24 (0.19–0.31)	<0.001
Adjusted OR_2_ (95% CI) ^b^	1.00	0.56 (0.45–0.70)	0.49 (0.39–0.62)	0.26 (0.20–0.33)	<0.001
PL plus PLP					
Median (nmol/L)	11.81	18.80	26.90	50.37	-
Cases/controls	403/313	370/311	271/311	189/310	-
Crude OR (95% CI)	1.00	0.92 (0.75–1.15)	0.68 (0.54–0.84)	0.47 (0.38–0.60)	<0.001
Adjusted OR_1_ (95% CI) ^a^	1.00	0.97 (0.78–1.22)	0.72 (0.57–0.91)	0.48 (0.38–0.61)	<0.001
Adjusted OR_2_ (95% CI) ^b^	1.00	0.97 (0.78–1.22)	0.73 (0.58–0.92)	0.51 (0.40–0.66)	<0.001
PAr					
Median	0.28	0.45	0.64	1.05	-
Cases/controls	161/311	250/312	341/311	481/311	-
Crude OR (95% CI)	1.00	1.54 (1.20–1.99)	2.12 (1.66–2.71)	3.00 (2.36–3.79)	<0.001
Adjusted OR_1_ (95% CI) ^a^	1.00	1.57 (1.20–2.04)	2.16 (1.67–2.78)	3.00 (2.34–3.85)	<0.001
Adjusted OR_2_ (95% CI) ^b^	1.00	1.52 (1.16–1.99)	2.16 (1.66–2.80)	2.90 (2.25–3.75)	<0.001

^a^ Adjusted for age, sex, BMI, education level, income level, marital status, smoking status, occupational activity, and MET-h/week. ^b^ Additionally adjusted for multivitamin supplement use, protein intake, total energy intake, vitamin B_2_ intake, folate intake, vitamin B_12_ intake, and methionine intake.

**Table 4:** Odds ratios and 95% confidence intervals of colorectal cancer according to quartiles of serum PLP, PL plus PLP, and PAr values stratified by sex and smoking status.

	Q1	Q2	Q3	Q4	*P*_trend_	*P*_interaction_
PLP						
Male						0.001
Median (nmol/L)	3.71	8.71	14.37	31.18		
Cases/controls	361/169	155/170	104/170	55/169		
Adjusted OR (95% CI) ^a^	1.00	0.46 (0.34–0.64)	0.30 (0.21-0.42)	0.15 (0.10–0.22)	<0.001	
Female						
Median (nmol/L)	2.77	6.16	11.36	26.89		
Cases/controls	201/142	138/142	149/142	70/141		
Adjusted OR (95% CI) ^a^	1.00	0.67 (0.48–0.94)	0.77 (0.55–1.07)	0.39 (0.27–0.57)	<0.001	
Never smokers						0.008
Median (nmol/L)	3.00	6.85	12.60	28.45		
Cases/controls	290/199	183/204	186/208	84/209		
Adjusted OR (95% CI) ^b^	1.00	0.59 (0.45–0.79)	0.63 (0.47–0.83)	0.30 (0.22–0.42)	<0.001	
Regular smokers						
Median (nmol/L)	3.61	8.64	14.06	27.47		
Cases/controls	182/68	80/73	37/66	26/63		
Adjusted OR (95% CI) ^b^	1.00	0.50 (0.31–0.80)	0.26 (0.15–0.46)	0.14 (0.08–0.26)	<0.001	
PL plus PLP						
Male						0.791
Median (nmol/L)	11.57	18.47	25.71	48.47		
Cases/controls	215/169	201/170	149/170	110/169		
Adjusted OR (95% CI) ^a^	1.00	1.11 (0.80–1.53)	0.76 (0.54–1.06)	0.53 (0.37–0.75)	<0.001	
Female						
Median (nmol/L)	12.10	19.26	28.24	52.66		
Cases/controls	188/144	169/141	122/141	79/141		
Adjusted OR (95% CI) ^a^	1.00	0.87 (0.62–1.20)	0.67 (0.48–0.95)	0.46 (0.31–0.67)	0.001	
Never smokers						
Median (nmol/L)	12.00	19.06	27.65	51.11		0.947
Cases/controls	244/201	222/210	168/205	109/204		
Adjusted OR (95% CI) ^b^	1.00	0.86 (0.65–1.14)	0.70 (0.52–0.94)	0.48 (0.35–0.66)	<0.001	
Regular smokers						
Median (nmol/L)	11.59	18.27	25.97	48.60		
Cases/controls	111/66	97/68	70/71	47/65		
Adjusted OR (95% CI) ^b^	1.00	1.26 (0.76–2.07)	0.85 (0.50–1.42)	0.47 (0.27–0.82)	0.006	
PAr						
Male						0.001
Median	0.28	0.43	0.61	1.00		
Cases/controls	82/169	95/170	200/170	298/169		
Adjusted OR (95% CI) ^a^	1.00	1.19 (0.80–1.79)	3.01 (2.06–4.39)	3.86 (2.68–5.57)	<0.001	
Female						
Median	0.27	0.48	0.69	1.09		
Cases/controls	79/142	154/142	142/141	183/142		
Adjusted OR (95% CI) ^a^	1.00	1.90 (1.30–2.77)	1.77 (1.20–2.59)	2.35 (1.62–3.42)	<0.001	
Never smokers						0.018
Median	0.28	0.46	0.67	1.07		
Cases/controls	100/203	184/205	192/213	267/199		
Adjusted OR (95% CI) ^b^	1.00	1.72 (1.24–2.38)	1.78 (1.29–2.46)	2.69 (1.96–3.70)	<0.001	
Regular smokers						
Median	0.27	0.44	0.61	0.98		
Cases/controls	41/75	37/65	103/63	144/67		
Adjusted OR (95% CI) ^b^	1.00	1.04 (0.56–1.95)	4.09 (2.30–7.27)	4.29 (2.46–7.47)	<0.001	

^a^ Adjusted for age, BMI, education level, income level, marital status, smoking status, occupational activity, MET-h/week, multivitamin supplement use, protein intake, total energy intake, vitamin B_2_ intake, folate intake, vitamin B_12_ intake, and methionine intake. ^b^ Adjusted for age, sex, BMI, education level, income level, marital status, occupational activity, MET-h/week, multivitamin supplement use, protein intake, total energy intake, vitamin B_2_ intake, folate intake, vitamin B_12_ intake, and methionine intake.

**Table 5:** Odds ratios and 95% confidence intervals of colorectal cancer according to quartiles of serum PLP, PL plus PLP, and PAr values by cancer site.

	Colon Cancer (*n* = 762)		Rectal Cancer (*n* = 471)		*P*_heterogeneity_
	Cases/Controls	Adjusted OR (95% CI) ^a^	Cases/Controls	Adjusted OR (95% CI) ^a^	
PLP					0.051
Q1	365/311	1.00	197/311	1.00	
Q2	170/312	0.49 (0.38–0.64)	123/312	0.69 (0.52–0.92)	
Q3	144/312	0.43 (0.33–0.56)	109/312	0.61 (0.45–0.83)	
Q4	83/310	0.26 (0.19–0.35)	42/310	0.25 (0.17–0.37)	
*P*_trend_		<0.001		<0.001	
PL plus PLP					0.994
Q1	251/313	1.00	152/313	1.00	
Q2	228/312	1.00 (0.77–1.29)	142/311	0.95 (0.71–1.27)	
Q3	166/311	0.72 (0.55–0.94)	105/311	0.75 (0.55–1.02)	
Q4	117/310	0.52 (0.39–0.69)	72/310	0.51 (0.36–0.71)	
*P*_trend_		<0.001		<0.001	
PAr					0.180
Q1	97/311	1.00	64/311	1.00	
Q2	141/312	1.39 (1.01–1.91)	108/312	1.66 (1.15–2.39)	
Q3	212/311	2.16 (1.60–2.93)	130/311	2.09 (1.46–2.98)	
Q4	312/311	3.07 (2.29–4.11)	169/311	2.48 (1.75–3.51)	
*P*_trend_		<0.001		<0.001	

^a^ Adjusted for age, sex, BMI, education level, income level, marital status, smoking status, MET-h/week, occupational activity, multivitamin supplement use, protein intake, total energy intake, vitamin B_2_ intake, folate intake, vitamin B_12_ intake, and methionine intake.

## Data Availability

The data that support the findings of our study are available from the corresponding author upon reasonable request. The authenticity of this article has been validated by uploading the key raw data onto the Research Data Deposit public platform (https://www.researchdata.org.cn/, accessed on 12 May 2020), with the approval RDD number as RDDA2022261257.
